# Supplementary material for: The impact of working hours on pregnancy intention in childbearing-age women in Korea, the country with the world’s lowest fertility rate
Source: PLoS One. 2023 Jul 19;18(7):e0288697. doi: 10.1371/journal.pone.0288697 (PMC10355408; doi:10.1371/journal.pone.0288697)
Supplement: Attachment — Submitted filename: Reviewer Comments_Akinsolu.docx [file pone.0288697.s002.docx]

Title of article reviewed:

The Impact of Working Hours on Pregnancy Intention in Women of Childbearing Age in Korea, the Country with the World's Lowest Fertility Rate

General Overview:

This study delves into the factors influencing pregnancy intentions among women of reproductive age in Korea, utilizing data from the Korean National Health and Nutrition Examination Survey (KNHANES). To analyze the characteristics of pregnant women and identify critical factors related to pregnancy intentions, the authors employed propensity score matching and the XGBoost machine learning model. The study's significant finding indicates that the number of weekly working hours substantially impacts pregnancy intentions. Specifically, women working between 35-45 hours per week exhibit higher odds of desiring pregnancy. Given the Korean context, where individuals tend to work excessively long hours, often surpassing the standard 40-hour workweek, these work patterns may deter or impede pregnancy intentions, affecting the nation's fertility rate.

Consequently, the research urges stricter monitoring of working hours and proposes promoting telecommuting for women in their childbearing years to bolster Korea's fertility rate. Beyond Korea, this study's findings possess broader implications for understanding the relationship between women's work hours and their intentions to conceive worldwide. It can influence workplace policies, particularly in countries with similar work cultures to Korea, by advocating for more flexible work hours or telecommuting to address demographic challenges associated with low birth rates.

Minor Comments:

Introduction:

Line 44: The acronym "OECD" should be defined before its usage.

Method:

Line 85: It is advisable to define "those not of childbearing age – 35,583."

Line 91-94: This section should be under "Ethical Considerations."

Line 96: The current title for this section is not appropriate.

Lines 97-103: This section should be titled "Statistical Analysis."

Lines 106-107: I recommend rewriting this section, as it appears to be mixed with the results. For example, lines 106-107 state, "The descriptive analysis of baseline characteristics and linear regression revealed a significant association among birth year, age, and participant characteristics."

Results:

Line 190-191: Please clarify this sentence: "Household income tended to increase with age, with the highest level observed among participants in the 40–49 age group."

Discussion:

Line 303-304: Reference 22 refers to another study that discusses similar findings. I suggest rephrasing the sentence to convey the message accurately.

Conclusion:

Lines 397-406: The conclusion should shed light on the significant impact of the study findings. Showing the results in this section is not appropriate. Please rephrase/rewrite the conclusion.
