# Supplementary material for: The impact of working hours on pregnancy intention in childbearing-age women in Korea, the country with the world’s lowest fertility rate
Source: PLoS One. 2023 Jul 19;18(7):e0288697. doi: 10.1371/journal.pone.0288697 (PMC10355408; doi:10.1371/journal.pone.0288697)
Supplement: Attachment — Submitted filename: PONE-D-23-12865.docx [file pone.0288697.s003.docx]

Reviewer’s Comments

Manuscript title:  **Impact of working hours on pregnancy intention in childbearing-age women in Korea, the country with the world’s lowest fertility rate**

Comments: the aim of the manuscript was well spelt out, and methods will outlined. The progression in statistical such as: propensity score matching, XGBoost machine learning model,cluster analysis and regression models (pages 12-15) were used with ‘R’. In conclusion, the study was clear of the factors (working hours and expansion of telecommuting) for increasing fertility rate among childbearing-age 42 women in Korea.

There was no major issues, however, minor issues in discussion: Line 192-196 should be well linked to the findings as reported in line 297-302. Previous studies should be inferred as to whether it support your findings or not.

In summary, there is the need to relate your findings to previous research in specific terms.

The use of English is critical . for example you cannot be suggesting policy implementation (as in line 334), and you use MUST (line 335)

Line 334-339 can be rephrased, no need to mention linear regression since you are proposing policy implementation and findings

Line 361-362 not clear (what is father’s situation)

Line 377-378 can be rephrased (However, this is the first statistical analysis study to examine the association between working hours and fertility among women of childbearing-age),

To the best of our understanding …………………………………………in Korea perhaps

Conclusion: Too long, don’t discuss, main finding and suggestion

**Recommendation**

The study is sound, the context is good, however language can be improved upon. I suggest that authors should revise the language to improve the text.

- Strongly recommended with minor corrections
